# Colloidal zinc oxide-copper(I) oxide nanocatalysts for selective aqueous photocatalytic carbon dioxide conversion into methane

**DOI:** 10.1038/s41467-017-01165-4

**Published:** 2017-11-07

**Authors:** Kyung-Lyul Bae, Jinmo Kim, Chan Kyu Lim, Ki Min Nam, Hyunjoon Song

**Affiliations:** 1 Department of Chemistry, Korea Advanced Institute of Science and Technology, and Center for Nanomaterials and Chemical Reactions, Institute for Basic Science (ibs), Daejeon, 34141 Republic of Korea; 2 Department of Chemistry, Mokpo National University, Jeonnam, 58554 Republic of Korea

## Abstract

Developing catalytic systems with high efficiency and selectivity is a fundamental issue for photochemical carbon dioxide conversion. In particular, rigorous control of the structure and morphology of photocatalysts is decisive for catalytic performance. Here, we report the synthesis of zinc oxide-copper(I) oxide hybrid nanoparticles as colloidal forms bearing copper(I) oxide nanocubes bound to zinc oxide spherical cores. The zinc oxide-copper(I) oxide nanoparticles behave as photocatalysts for the direct conversion of carbon dioxide to methane in an aqueous medium, under ambient pressure and temperature. The catalysts produce methane with an activity of 1080 μmol g_cat_^−1^ h^−1^, a quantum yield of 1.5% and a selectivity for methane of >99%. The catalytic ability of the zinc oxide-copper(I) oxide hybrid catalyst is attributed to excellent band alignment of the zinc-oxide and copper(I) oxide domains, few surface defects which reduce defect-induced charge recombination and enhance electron transfer to the reagents, and a high-surface area colloidal morphology.

## Abstract (web summary)

Photocatalytic reduction and oxidation reactions, involving multiple electrons and operating in tandem, are extremely challenging to achieve. Here, with a hybrid structure of ZnO and Cu_2_O, the authors report photocatalytic carbon dioxide reduction to methane with >99% selectivity using electrons from water.

## Introduction

There has been intensive research on direct carbon dioxide (CO_2_) conversion reactions via photochemical, electrochemical, and biological approaches^1–3^. A photochemical method using sun light in aqueous solutions is regarded as a leading potential approach due to the prospect of using free and plentiful solar energy without damaging the environment^4–6^. Titanium dioxide (TiO_2_) is a representative photocatalytic material for this purpose, due to its effective charge separation ability, abundance, and low environmental toxicity^7, 8^. The addition of co-catalysts such as platinum (Pt) and copper (Cu) can enhance the catalytic activity^9–11^. However, these TiO_2_-based hybrid catalysts mostly generate hydrogen (H_2_) rather than carbon species from carbonated water^12^, because the electrochemical reduction potentials of water to H_2_ (−0.41 V vs. normal hydrogen electrode (NHE)) and CO_2_ to reduced species (−0.58 to −0.24 V vs. NHE) are in a similar range^9^. Consequently, a novel strategy for increasing selectivity would be helpful for enhancing CO_2_ conversion reactions.

Copper oxides are p-type semiconductors with narrow bandgaps (CuO, *E*_g_ = 1.35 to 1.7 eV; Cu_2_O, *E*_g_ = 1.9 to 2.2 eV) and have been employed in pigments, solar cells, electrodes, and catalysts for organic reactions^13, 14^. In particular, their favorable light absorption in the visible range enables copper oxides to be photocatalytic materials. The formation of hybrids with TiO_2_ can form p–n type junctions, which exhibit better charge separation and enhanced activity for photocatalytic CO_2_ reduction^13^. Schaak et al.^15^ deposited TiO_2_ onto Cu_3_N nanocubes at high temperature to yield hollow TiO_2−*x*_N_*x*_-CuO nanocubes, which showed high conversion of CO_2_ to CH_4_^15^. Ye et al.^16^ synthesized porous TiO_2_-Cu_2_O nanojunction materials, which exhibited a large enhancement in CH_4_ evolution activity. Although the proper combination of semiconductor and co-catalyst is essential, the structure and morphology (e.g. size, shape, and surface structure of each domain and their interfaces) is also critical in determining the catalytic properties. Rigorous control of these factors is critical for designing a photocatalyst with optimal performance^17^.

Here, we select the combination of Zn(II) oxide and Cu(I) oxide for effective photocatalytic CO_2_ conversion. Zn-Cu oxides are known from their use as photocatalysts for dye degradation^18, 19^. We expect that Zn and Cu oxides will also be an excellent photocatalyst for CO_2_ reduction, due to the fact that CO_2_ species are readily adsorbed on the surface sites of metal oxides^20, 21^. Guided by this inspiration, we are able to successfully grow Cu_2_O single-crystalline nanocubes on ZnO surfaces, generating a ZnO-Cu_2_O hybrid nanostructure with well-defined surface structures. In the absence of any additional sacrificial reagents, CO_2_ reduction occurs in neutral carbonated water using the colloidal ZnO-Cu_2_O catalyst. The resulting CH_4_ production rate is 1080 μmol g_cat_^−1^ h^−1^, which is one of the highest activities reported thus far in an aqueous medium. The estimated quantum efficiency (QE) is 1.5%, and the selectivity of CH_4_ production exceeds 99%, whereas a control experiment with a TiO_2_-Cu_2_O catalyst mainly generates H_2_.

## Results

### Synthesis and characterization of ZnO-Cu_2_O hybrid nanoparticles

**Fig. 1:**
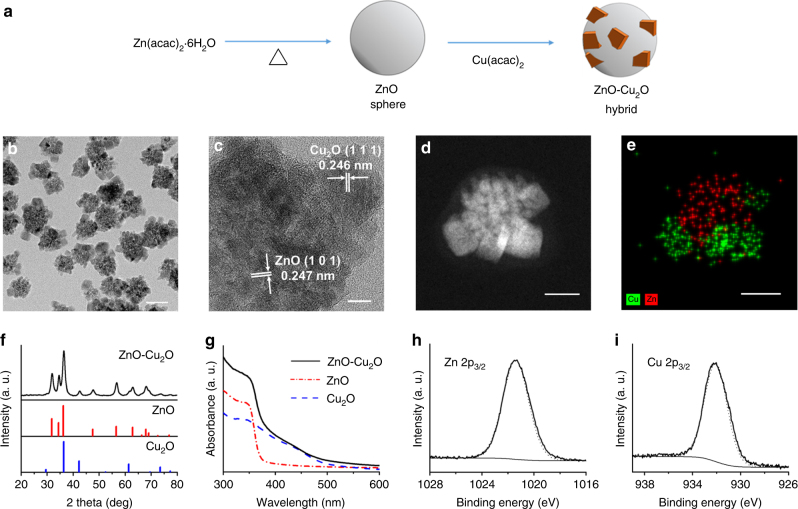
Synthesis and characterization of the ZnO-Cu_2_O hybrid nanoparticles. **a** Synthesis of ZnO-Cu_2_O hybrid nanoparticles via a two-step in situ process. **b** TEM image of ZnO-Cu_2_O nanoparticles. **c** HRTEM and **d** STEM images, and **e** elemental mapping of an individual ZnO-Cu_2_O nanoparticle. The bars represent **b** 50, **c** 5, and **d**, **e** 20 nm. **f** XRD and **g** UV–Vis spectra of ZnO-Cu_2_O hybrid nanoparticles. XPS spectra of ZnO-Cu_2_O hybrid nanoparticles in the regions of **h** Zn 2p_3/2_ and **i** Cu 2p_3/2_

ZnO-Cu_2_O hybrid nanoparticles were synthesized via a two-step process in a single batch. ZnO spheres were formed through a polyol process in the presence of PVP (poly(vinyl pyrrolidone)) behaving as a surfactant. After the complete hydrolysis of Zn precursors, a Cu precursor solution was added in situ to the reaction mixture and heated for an additional 5 min. Rapid cooling and separation yielded ZnO-Cu_2_O nanoparticles (Fig. 1a)^22^. The transmission electron microscopy (TEM) image in Fig. 1b shows that each particle has an isolated structure containing multiple cubic shapes attached to a spherical core. The average diameter of the spherical cores was 40 ± 7 nm, and that of the cubic domains 18 ± 3 nm. The high-resolution TEM (HRTEM) image in Fig. 1c shows that the spherical core is actually an aggregate of small single-crystalline domains, in which the average size of each domain is estimated to be 7 ± 1 nm. A cubic domain attached to the core is also single crystalline. The distances between adjacent lattice fringe images are nearly identical over all domains, 0.247 nm in the core and 0.246 nm in the cubic domain. The scanning transmission electron microscopy (STEM) image in Fig. 1d clearly shows that an individual particle is composed of a spherical aggregate of small particulates in the core, with multiple rectangular domains bound to it. The elemental mapping image by energy dispersive X-ray spectroscopy (EDX) in Fig. 1e indicates that Zn and Cu components are completely separated, with Zn located in the spherical core and Cu in the cubic domains.

X-ray diffraction (XRD) data in Fig. 1f show that the pattern is an exact sum of the diffractions from hexagonal wurtzite ZnO (red, JCPDS #36-1451) and primitive cubic Cu_2_O (blue, JCPDS #77-0199). The single-crystalline domain size of the ZnO cores is estimated to be 7.9 nm from the FWHM of ZnO(101) peak using the Scherrer equation, in good agreement with the size measured by the HRTEM image. The ultraviolet (UV)–visible (Vis) spectrum of the ZnO-Cu_2_O nanoparticles in Fig. 1g is also a linear combination of those for ZnO and Cu_2_O nanoparticles. The band gap energies of the ZnO and Cu_2_O domains are 3.3 eV and 2.3 eV, respectively, estimated using Tauc plots of the UV–Vis spectra (Supplementary Fig. 1)^23^. X-ray photoelectron spectroscopy (XPS) in the Zn 2p_3/2_ region shows that a single peak at 1021.4 eV is assignable to Zn(II) (Fig. 1h). In particular, the spectrum in the Cu 2p_3/2_ region shows a single symmetric peak at 932.1 eV, indicating that there was no formation of Cu(II) during the synthesis (Fig. 1i). These observations confirm that the product is ZnO-Cu_2_O hybrid nanoparticles with ZnO in the cores and Cu_2_O in the cubic domains.

It is known that the Zn precursors were hydrolyzed in an alcoholic medium to generate Zn alcoxides, which were transformed into ZnO nanocrystalline seeds by dehydration at high temperature. The small seeds were simultaneously aggregated to yield large spheres via an oriented attachment mechanism^24^. Then, the Cu precursors were hydrolyzed and reduced to Cu^+^ and formed Cu_2_O on the ZnO surface. It is noted that the distance (0.247 nm) of adjacent lattice fringe images in the sphere in Fig. 1c matches the distance of ZnO(101) planes. It is nearly identical to the distance of lattice fringes in the cubic domain of 0.246 nm, assignable to the distance of Cu_2_O(111). This low lattice mismatch may lead to the direct growth of Cu_2_O on the ZnO surface forming good junctions. The crystal structure of Cu_2_O is primitive cubic so the Cu_2_O domains grow to generate cubic-type morphology by preferential adsorption of PVP on the Cu_2_O(100) surface.

### Photocatalytic CO_2_ conversion using ZnO-Cu_2_O hybrid catalysts

**Fig. 2:**
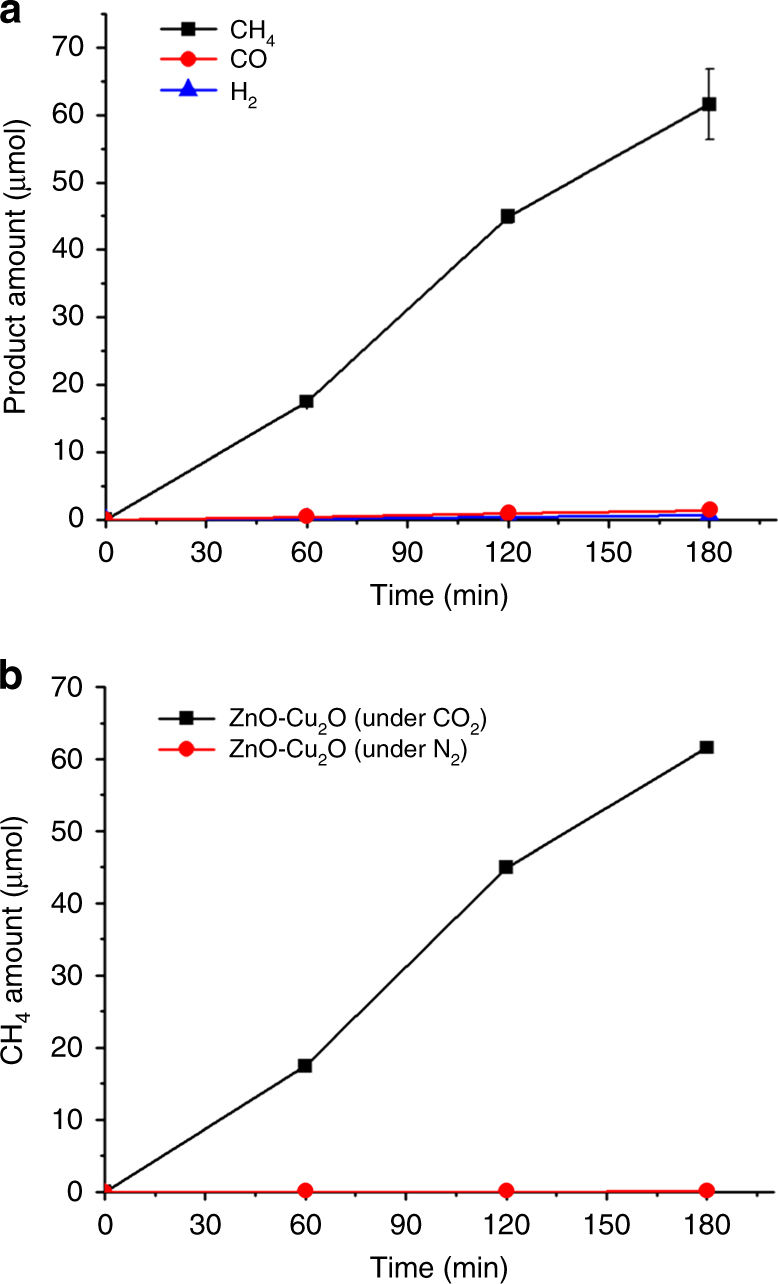
Photocatalytic CO_2_ conversion experiments. **a** Amounts of CH_4_ (black), CO (red), and H_2_ (blue) production, and **b** amounts of CH_4_ production under CO_2_ saturation (black) and N_2_ bubbling (red) conditions using the ZnO-Cu_2_O catalysts as a function of the irradiation time. The reaction conditions (**a**, **b**) were catalyst amount 19 mg, pH = 7.4, and *λ* > 200 nm. The error bars were obtained from three independent experiments

The photocatalytic CO_2_ conversion reaction was conducted using our well-defined ZnO-Cu_2_O hybrid nanoparticles in an aqueous medium. ZnO is an n-type metal oxide semiconductor with a large band gap (3.2 to 3.3 eV) with a low dielectric constant and high electron mobility, compared to those of TiO_2_^25, 26^. For dye degradation reactions, ZnO catalysts show better photocatalytic activity than TiO_2_ counterparts under the irradiation of UV–Vis light^27^. A few examples of ZnO-Cu_2_O heterostructures exhibited enhancement of dye degradation through the formation of p-n junctions^18, 19, 28^. In the present experiments, the pH of the reaction medium was fixed to 7.4 by the addition of perchloric acid. At this pH, the ZnO-Cu_2_O hybrid catalysts were stable to assess the photocatalytic reactions by prolonged UV–Vis irradiation. The CO_2_ saturation in the aqueous medium was achieved using a 0.2 M Na_2_CO_3_ solution stirred under CO_2_ pressure of 2.6 bar for 40 min^29^. After release of the pressure, CO_2_ bubbling was continued at ambient pressure and temperature. By the irradiation of light using a 300 W Xe lamp, only two chemicals, CH_4_ and CO, were detected as gaseous products using gas chromatography. CH_4_ was the primary product with the amount of 62 μmol for 3 h (Fig. 2a), equating to catalytic activity of 1080 μmol g_cat_^−1^ h^−1^ with respect to the total amount (19 mg) of the ZnO-Cu_2_O catalyst used in this reaction. Remarkably, the amounts of CO and H_2_ generation were only 1.4 and 0.7 μmol, respectively, which means that the selectivity of CH_4_ production was higher than 99%. To prove the CH_4_ production was not from organic residues, a control experiment was carried out under N_2_ atmosphere. By irradiation with light for 3 h, the catalyst showed a negligible CH_4_ production of 8.4 × 10^−3^ μmol (red, Fig. 2b). No reaction occurred in the absence of irradiation or catalyst, meaning that the CH_4_ production actually originated from photocatalytic CO_2_ reduction in the presence of the ZnO-Cu_2_O catalyst. The reaction in the presence of ^13^CO_2_ was also carried out (Supplementary Fig. 2). Based on a signal at *m*/*e* = 17, assignable to the ^13^CH_4_ peak in the gas chromatography–mass spectrometry (GC–MS) chromatogram when ^13^CO_2_ and Na_2_^13^CO_3_ were used, the percentage of CH_4_ directly generated from CO_2_ was estimated to be 88% during the reaction.

Eq. 1 was used to estimate the QE of CO_2_ photoconversion to CH_4_:^30^

1 $${\rm{QE}}\left( {\rm{\% }} \right) = \frac{{8 \times {\rm{Number}}\,{\rm{of}}\,{\rm{C}}{{\rm{H}}_4}\,{\rm{molecules}}}}{{{\rm{Number}}\,{\rm{of}}\,{\rm{incident}}\,{\rm{photons}}}} \times 100$$

It is noted that eight electrons are required for the production of one CH_4_ molecule from CO_2_. The number of photons was calculated using the wavelength region between 200 to 540 nm based on the UV–Vis absorption of the catalysts (Fig. 1g) and the intensity of the incident light. The QE from photons to CH_4_ molecules was estimated to be 1.5%.

**Fig. 3:**
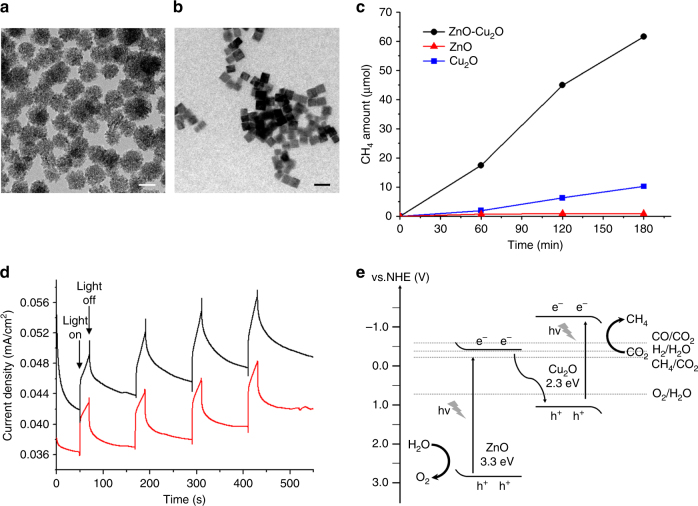
Control experiments with the ZnO and Cu_2_O nanoparticles and mechanistic study of the ZnO-Cu_2_O catalysts. TEM images of **a** ZnO spheres and **b** Cu_2_O nanocubes. The scale bars represent 50 nm. **c** Amount of CH_4_ production using ZnO-Cu_2_O (black) catalysts, and ZnO (red) and Cu_2_O (blue) nanoparticles under the conditions with catalyst amount fixed to 19 mg, pH = 7.4, and *λ* > 200 nm. **d** Photoresponse data of the ZnO-Cu_2_O catalyst deposited on a FTO electrode at a potential of −0.45 V vs. Ag/AgCl in a phosphate buffer by the irradiation of UV–visible (black) and visible (red) light using a cutoff filter (*λ* > 425 nm). **e** Band alignment and proposed electron transfer mechanism of the ZnO-Cu_2_O hybrid catalysts

To ensure that the ZnO-Cu_2_O hybrid structure is critical in CH_4_ production, ZnO spheres and Cu_2_O cubes with similar size and morphology were prepared (Fig. 3a, b) and employed for photocatalytic CO_2_ conversion. Under the experimental conditions, the activity of the ZnO spheres was estimated to be 15 μmol g_cat_^−1^ h^−1^, and that of the Cu_2_O particles was 180 μmol g_cat_^−1^ h^−1^, for CH_4_ production (Fig. 3c). The lifetime of photogenerated electrons was directly measured by means of time-correlated single photon counting (TCSPC). The decay of transient absorption was measured at 620 nm, which corresponds to transition between the energy bands at the interface^31^. The photoexcited electron lifetime of ZnO-Cu_2_O nanoparticles (*τ*_1/2_ = 837.1 ps) is large, compared to those of ZnO (*τ*_1/2_ = 491.4 ps), and Cu_2_O (*τ*_1/2_ = 206.5 ps) nanoparticles (Supplementary Fig. 3). This may be attributed to the interfacial states trapping electrons, which reduces the rate of charge recombination from the conduction band to the valence band of ZnO and facilitate tunneling to the valence band of Cu_2_O^32^. The photo-response of the ZnO-Cu_2_O catalysts was measured during irradiation with UV–Vis and visible light (>425 nm). The catalyst deposited on a FTO electrode generated cathodic photocurrents at an applied potential of −0.45 V vs. Ag/AgCl in a phosphate buffer, implying a p-type characteristic of the Cu_2_O domains. The electrode generated almost identical photocurrents under UV–Vis and visible light irradiation, indicating that the visible light absorption in the Cu_2_O domains is critical to generate photoelectrons (Fig. 3d).

The CH_4_ production rates were also measured using ZnO-Cu_2_O catalysts synthesized from various ratios of the Zn/Cu precursors, but the activities were inferior to that of the optimized catalyst (Supplementary Fig. 4). This is because either the Cu_2_O domains were not fully grown on the ZnO surface, or the resulting catalyst was not uniform in its morphology. This indicates that the catalyst structure is an essential factor to maximize the catalytic performances.

### Mechanistic aspects of CO_2_ conversion reactions

A mechanism of CO_2_ reduction is proposed based on these experimental results. The ZnO-Cu_2_O hybrid catalyst absorbs both UV and visible light corresponding to the bandgaps of 3.3 eV for the ZnO and 2.3 eV for Cu_2_O domains (Fig. 1g). Well-defined domain structures are expected to induce an appropriate bandgap alignment as depicted in Fig. 3e ^4, 33–35^. In the Z-scheme mechanism, the effective electron transfer from the conduction band of ZnO to the valence band of Cu_2_O domains leads to long-lived charge separation states with the excited electrons at the conduction band of the Cu_2_O domain and the holes at the valence band of the ZnO domain. The excited electrons are eventually transferred to the surface-adsorbed CO_2_, and the holes are transferred to water molecules. With this mechanism, high activity of the ZnO-Cu_2_O catalyst system can be explained by the following aspects. First, ZnO has a lower dielectric constant and a higher electron mobility than TiO_2_^25–27^, which causes a low electron-hole recombination rate in photochemical reactions. The valence band edge energy of ZnO (2.8 eV vs. NHE) is far lower than the water oxidation potential (0.82 V vs. NHE), which overcomes the large overpotential commonly required for water oxidation reactions. Cu_2_O is also superior to CuO for CO_2_ reduction by water, due to its large bandgap (2.4 eV) with a high-conduction band edge energy (−1.4 V vs. NHE) compared to that of CuO (−0.8 V vs. NHE)^4, 36^. It is also significantly higher than the reduction potentials of CO_2_ to other reduced products (−0.24 ~ −0.58 V vs. NHE)^5, 9^, supplying a sufficient amount of energy to the reactants. These bandgap energies render the combination of ZnO-Cu_2_O a good fit with the ideal band diagram for facile CO_2_ reduction (Fig. 3e). Second, the formation of uniform domain structures facilitates electron and hole transfers to the reagents. When a photocatalyst is immersed in water, charge transfer occurs at the semiconductor-solution interface due to the equilibration of electron density between two phases^37, 38^. The net result is the formation of an electrical field at the semiconductor surface. In the case of n-type semiconductors (ZnO), when photogenerated electron-hole pairs form in the space charge region, this leads to hole transfer to the surface and water oxidation. Similarly, photogenerated electrons move to the surface and reduce CO_2_ in p-type semiconductors (Cu_2_O). In general, surface defects result in the formation of defect energy levels, and trap the charges, lowering the quantum yields^39^. In our ZnO-Cu_2_O hybrid nanoparticles, the cubic Cu_2_O domains are covered by the defect-less Cu_2_O(100) facets, and the ZnO is composed of single-crystalline domains as large as 8 nm in diameter. These have fewer surface defects than any other Zn-Cu structures^18, 19, 28^, and this enhances charge transfer to the reagents. Third, the discrete morphology of the nanoparticles, by which a colloidal dispersion is readily formed in aqueous medium, is advantageous in terms of higher surface area than those of large powders or aggregates. CO_2_ molecules should be continuously adsorbed onto the surface sites of the Cu_2_O domains, and protons in water also approach the reaction sites. Therefore, the high surface area resulting from the colloidal morphology is critical for the absorption of both reactants needed to achieve high activity.

The other mechanism, double charge transfer, which includes electron transfer from the conduction band of Cu_2_O to ZnO domains and hole transfer from the valance band of ZnO to Cu_2_O, has also been proposed in several photoreduction systems^16, 31, 39^. However, in our catalysts, the CH_4_ production of the pure ZnO aggregates was negligible, while the pure Cu_2_O nanoparticles showed a significant activity (Fig. 3c), indicating that the Cu_2_O domains are main active sites for CO_2_ reduction. In the aspect of band edge energies, the Z-scheme mechanism in Fig. 3e is more reasonable to provide large overpotentials of both CO_2_ reduction and water oxidation reactions, which the double charge transfer mechanism cannot offer. To suggest the proper photophysical mechanism, the reaction was carried out by irradiation with visible light (UV cutoff filter *λ* > 420 nm) under the present conditions. The CH_4_ production was almost negligible during the reaction, and the surface state of the catalyst was unchanged after the reaction. After the removal of the cutoff filter, CH_4_ was generated with activity similar to that of the original experiment at a fixed light intensity of 0.59 Wcm^−2^ (Supplementary Fig. 5). This result indicates that the excitation of electrons in the ZnO domain is critical to activate the catalyst, and a Z-scheme is a more reliable reaction mechanism for our catalytic system.

For the issue of selectivity, this photocatalytic system provides sufficient energy, due to the Z-scheme, to provide photoexcited electrons at a high energy level for CO_2_ reduction. It is known that the products are highly dependent upon the relative energy levels of intermediates^4, 5, 40^. Gattrell and many other researchers suggested that the radical anion of CO_2_ is adsorbed on the metal surface and forms a carboxylic radical, which converts to CO by the interaction with surface hydrogen radical^8, 41, 42^. According to the calculations, the rate determining step of the process is the hydrogenation of CO into the formyl radical, which strongly influences the product distribution. Cu has a strong binding strength for adsorbed intermediates and facilitates the hydrogenation. More specifically, it is reported that the intermediates are particularly stabilized on the Cu_2_O(100) surface^43^, which prevents the desorption of CO and allows efficient coupling with protons during the reaction. In the present reaction conditions, the reaction medium contains a high proton concentration at neutral pH and behaves as a rich hydrogen source that directly supplies protons^8, 41^. The resulting intermediates, such as formyl radicals or carbenes, are further hydrogenized to eventually produce CH_4_. To understand the reaction mechanism in detail further studies are required.

The counter reaction, oxidation, should be driven by the photogenerated holes at the same time. Mostly the holes were transferred to water molecules and led to oxygen evolution, which was detectable by GC, but the PVP adsorbed on the catalyst surface might also behave as a hole scavenger during the early stage of the photocatalytic reaction.

### Comparison to the TiO_2_(P25)-Cu_2_O hybrid catalysts

**Fig. 4:**
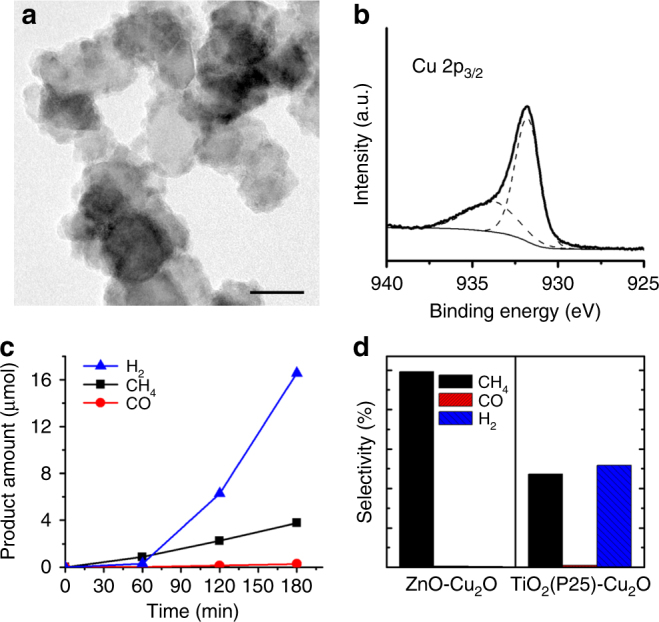
Comparison to the TiO_2_(P25)-Cu_2_O hybrid catalysts. **a** TEM image of the TiO_2_(P25)-Cu_2_O hybrid structure. The scale bar represents 20 nm. **b** XPS spectrum of the TiO_2_(P25)-Cu_2_O hybrid structure in the region of Cu 2p_3/2_. **c** Amounts of H_2_ (blue triangle), CH_4_ (black square), and CO (red circle) production using the TiO_2_(P25)-Cu_2_O catalysts as a function of the irradiation time. **d** Selectivity of gas products using ZnO-Cu_2_O (left) and TiO_2_(P25)-Cu_2_O (right) catalysts

For comparison, we synthesized a TiO_2_(P25)-Cu_2_O hybrid structure to investigate the composition and morphology effects vs. catalytic performance. The TiO_2_(P25)-Cu_2_O hybrids were synthesized via the reduction of the Cu precursors in the presence of commercial P25. A TEM image and EDX analysis indicate that the Cu domains were successfully deposited on P25 (Fig. 4a and Supplementary Fig. 6). The XPS data in the region of Cu 2p_3/2_ also indicates the presence of Cu species on the surface (Fig. 4b). Under the present reaction conditions of CO_2_ reduction by irradiation for 3 h, the quantity of gaseous products using the TiO_2_(P25)-Cu_2_O catalysts (9.5 mg) were 3.8, 0.28, and 17 μmol for CH_4_, CO, and H_2_, respectively. The activity for each product was estimated as 130, 10, and 580 μmol g_cat_^−1^ h^−1^ for CH_4_, CO, and H_2_, respectively, of which the total gas production was inferior to that using the ZnO-Cu_2_O catalyst (Fig. 4c). In particular, the TiO_2_(P25)-Cu_2_O catalysts generated H_2_ as a major product and CH_4_ as the second, whereas the ZnO-Cu_2_O catalysts showed 99% selectivity for CH_4_ (Fig. 4d). Regarding direct CO_2_ conversion, the ZnO-Cu_2_O catalysts are superior to TiO_2_(P25)-Cu_2_O for both reaction activity and selectivity.

### Stability of the ZnO-Cu_2_O catalyst

**Fig. 5:**
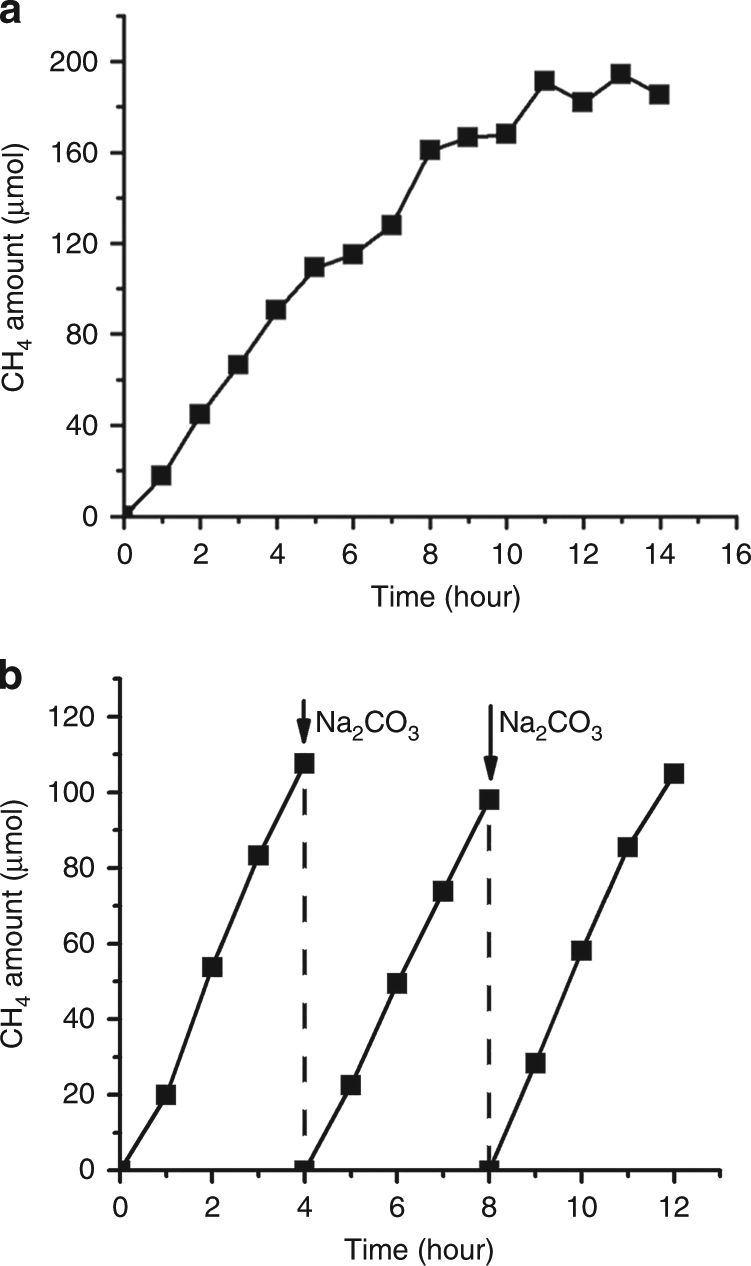
Stability experiment of the ZnO-Cu_2_O catalysts. **a** Amount of CH_4_ production using the ZnO-Cu_2_O catalysts as a function of the irradiation time up to 14 h. The reaction conditions were pH = 7.4 and *λ* > 200 nm. The CH_4_ amount was converted based on the catalyst amount fixed to 19 mg. **b** The amount of CH_4_ production under the identical reaction conditions except the change of the reaction medium at each 4 h reaction time

The durability of the ZnO-Cu_2_O catalyst was tested under the present reaction conditions. The CH_4_ production rate was constant under prolonged irradiation up to 8 h, and then dropped at over 11 h. (Fig. 5a). The reaction was carried out in a closed chamber; therefore, CO_2_ depletion in the reaction medium may be the main reason for this activity decrease (See Supplementary Information). To prove the catalyst stability, multiple reactions with repeated CO_2_ charging in the chamber were attempted. The reaction profile indicates that the CH_4_ production linearly increased for more than 4 h. At this period, the reaction was stopped, the catalyst particles were re-dispersed in a fresh reaction medium with 0.2 M Na_2_CO_3_, and additional reactions were carried out under identical conditions. This process was repeated one more time. In each trial, the amount of CH_4_ production linearly increased, and the reaction activity was nearly unchanged as shown in Fig. 5b. This implies that the catalyst stability was maintained over the reaction period of 12 h, when the fresh reaction medium was supplied. Instead of using the static reaction conditions inside the chamber, a continuous CO_2_ flow through the reaction mixture is a potential solution to enhance the catalyst stability.

### Comparison to other photocatalysts used for CO_2_ conversion

The catalytic performance of the ZnO-Cu_2_O hybrid catalyst is listed with those of other catalysts reported in the literature (Table 1). It is very difficult to provide a direct comparison to other CO_2_ reduction catalysts, due to different experimental conditions such as light source, reaction medium, and distinct products. However, the ZnO-Cu_2_O catalyst exhibits one of the highest activities and quantum yields among catalysts in aqueous media; sometimes two or three orders of magnitude higher than those of the other catalysts. The activity of the ZnO-Cu_2_O catalyst is comparable to the highest activities observed among solid catalysts under the conditions of high pressure CO_2_ and high temperature.

**Table 1:** Comparison of the reaction conditions and performances with other catalysts for photocatalytic CO_2_ reduction

Catalyst	Light source	Reaction medium	Products	Activity	Reference
ZnO-Cu_2_O	300 W Xe lamp	Saturated CO_2_ in water, 0.2 M Na_2_CO_3_	CH_4_	1080 μmol g_cat_^−1^ h^−1^ QE = 1.5%	This work
Colloidal CdS	Medium pressure arc lamp	Saturated CO_2_ in water, 0.1 M TMACl, 0.01 M hydroquinone	HCOOH, CH_2_O, glyoxylic acid	3.21 μmol g_cat_^−1^ h^−1^ for HCOOH QE = 0.125%	Grimshaw et al. (ref. ^44^)
NiO-InTaO_4_	Circular fluorescent lamp	Saturated CO_2_ in water, 0.2 M NaOH	CH_3_OH	2.8 μmol g_cat_^−1^ h^−1^ QE = 0.0045%	Wu et al. (ref. ^45^)
2.0% Cu/TiO_2_	8 W UV Hg lamp	Saturated CO_2_ in water, 0.2 M NaOH	CH_3_OH	19.75 μmol g_cat_^−1^ h^−1^ QE = 10.02%	Wu et al. (ref. ^46^)
Nafion/Pd-TiO_2_	300 W Xe lamp	Saturated CO_2_ in water, 0.2 M Na_2_CO_3_	CH_4_	45 μmol g_cat_^−1^ h^−1^	Choi et al. (ref. ^29^)
Ru(bpz)_3_^2+^/Ru	*λ* > 420 nm	Saturated CO_2_ in water/CH_3_CH_2_OH, 0.05 M NaHCO_3_, 0.17 M TEOA	CH_4_	QE = 0.04%	Willner et al. (ref. ^47^)
Pt-TiO_2_ thin film	400 W Xe lamp	CO_2_ and water flow of 3 mL min^−1^	CH_4_	1361 μmol g_cat_^−1^ h^−1^ QE = 2.6%	Biswas et al. (ref. ^11^)
AuCu-P25	1000 W Xe lamp (AM 1.5)	1.7 atm water saturated CO_2_, 60 °C	CH_4_	2200 μmol g_cat_^−1^ h^−1^	Garcia et al. (ref. ^48^)

## Discussion

ZnO-Cu_2_O hybrid nanoparticles were synthesized through the direct surface growth of Cu_2_O on ZnO spheres. The resulting nanoparticles have ZnO and Cu_2_O domains with few surface defects and well-defined junctions. Photochemical CO_2_ reduction reactions were carried out using the ZnO-Cu_2_O catalyst in an aqueous medium under ambient conditions. The catalyst exhibited a high reactivity of 1080 μmol g_cat_^−1^ h^−1^ with a QE of 1.5% and 99% selectivity for CH_4_. This performance for selective CH_4_ generation is attributed to the energetic match between the ZnO and Cu_2_O components, and their defect-less surface and junctions. These properties suppress charge recombination and enhance effective charge transfer. This strategy to design and synthesize well-defined nanostructures as colloidal forms could be expandable to other materials for photochemical reactions. It might also have a significant impact on the understanding of the mechanisms and key factors needed to achieve maximum catalytic performance.

## Methods

### Chemicals

Zinc(II) acetylacetonate hexahydrate (Zn(acac)_2_∙6H_2_O, 99.995%), 1,5-pentanediol (1,5-PD, 96%), poly(vinyl pyrrolidone) (PVP, *M*_w_ = 55,000), copper(II) acetylacetonate (Cu(acac)_2_, ≥ 99.95%), titanium (IV) oxide (P25, TiO_2_, 99.5%), sodium carbonate (Na_2_CO_3_, ≥ 99.0%), and perchloric acid (HClO_4_, 60%) were purchased from Sigma-Aldrich and used without further purification.

### Synthesis of ZnO-Cu_2_O hybrid nanoparticles

Zinc acetylacetonate hexahydrate (0.10 g, 0.40 mmol) and PVP (1.0 g, 9.0 mmol) were dissolved in 1,5-PD (50 mL) under inert conditions at 130 °C to ensure complete dissolution. The solution was heated to 225 °C for 6 min and allowed to stir for 3 min at the same temperature. Copper acetylacetonate (0.10 g, 0.40 mmol) was dissolved in 1,5-PD (5.0 mL) under an inert condition. The Cu precursor solution was added to the reaction mixture at 225 °C, followed by stirring for 5 min at the same temperature. After rapid cooling to room temperature using an ice bath, the product was separated by the addition of ethanol (60 mL) with the aid of centrifugation at 10,000 rpm. The precipitates were thoroughly washed with ethanol.

### Synthesis of ZnO spheres

Zinc acetylacetonate hexahydrate (0.10 g, 0.40 mmol) and PVP (1.0 g, 9.0 mmol) were dissolved in 1,5-PD (50 mL) under an inert condition at 130 °C to ensure complete dissolution. The solution was heated to 225 °C for 6 min and allowed to stir for 5 min at the same temperature. After rapid cooling to room temperature using an ice bath, the product was separated by the addition of ethanol (60 mL) with the aid of centrifugation at 10,000 rpm. The precipitates were thoroughly washed with ethanol.

### Synthesis of Cu_2_O nanocubes

PVP (1.0 g, 9.0 mmol) was dissolved in 1,5-PD (50 mL) under an inert condition at 130 °C to ensure the complete dissolution. Copper acetylacetonate (0.10 g, 0.40 mmol) was dissolved in 1,5-PD (5.0 mL) under inert conditions. This Cu precursor solution was added to the reaction mixture at 225 °C, followed by stirring for 5 min at the same temperature. After rapid cooling to room temperature using an ice bath, the product was separated by the addition of ethanol (60 mL) with the aid of centrifugation at 10,000 rpm. The precipitates were thoroughly washed with ethanol.

### Synthesis of TiO_2_(P25)-Cu_2_O hybrid nanoparticles

Titanium (IV) oxide (P25, 0.030 g, 0.40 mmol) and PVP (0.5 g, 4.5 mmol) were dissolved in 1,5-PD (50 mL) under inert conditions at 130 °C to ensure complete dissolution. Copper acetylacetonate (0.10 g, 0.40 mmol) was dissolved in 1,5-PD (5.0 mL) under an inert condition. The Cu precursor solution was added to the reaction mixture at 225 °C, followed by stirring for 5 min at the same temperature. After rapid cooling to room temperature using an ice bath, the product was separated by the addition of ethanol (60 mL) with the aid of centrifugation at 10,000 rpm. The precipitates were thoroughly washed with ethanol.

### Characterization

TEM images and energy dispersive X-ray diffraction (EDX) data of the nanoparticles were obtained by FEI Tecnai G2 F30 S-Twin (300 kV, KAIST), and HRTEM images and elemental mapping were obtained by FEI Titan cubed G2 60–300 (double Cs corrected, KAIST) transmission electron microscopes. Samples were prepared by dropping a few samples dispersed in ethanol on carbon-coated 200 mesh nickel grids (Ted Pella Inc.). XRD patterns of the samples were recorded on a Rigaku D/MAX-2500 diffractometer. X-ray photoelectron spectra (XPS) were obtained by K-alpha X-ray photoelectron spectroscopy (Thermo VG Scientific). UV–Vis spectra were measured on a UV-3600 UV-vis-NIR spectrophotometer (Dong-il Shimadzu Corp.). TCSPC was measured by a FL920 spectrometer (Edinburgh Instruments).

### Photocatalytic CO_2_ conversion experiments

The ZnO-Cu_2_O catalysts (19 mg) were dispersed in a 0.2 M Na_2_CO_3_ aqueous solution (20 mL), and the dispersion was neutralized to pH = 7.4 by the addition of HClO_4_. The reactor was a homemade quartz flask with a total volume of 41 mL. Supercritical-fluid grade CO_2_ gas was used to avoid any hydrocarbon contamination. To reach CO_2_ saturation in the reaction medium, the catalyst dispersion was stirred for 40 min in a high pressure chamber under a CO_2_ pressure of 2.6 bar. After the pressure release, CO_2_ gas was transferred to the quartz reactor and was additionally bubbled at ambient pressure and temperature for 30 min. Photocatalytic CO_2_ conversion was conducted by irradiation from a Xe lamp (300 W, Oriel) equipped with a 10 cm IR water filter. During the reaction, the gas product was collected using a needle-type probe passing through a sealed rubber septum. The gas samples were analyzed by thermal conductivity detector (TCD) and flame ionization detector (FID) equipped with a carboxen 1000 column (Supelco) via gas chromatography (YL6100 GC). Before the FID detector, a methanizer (500 mg Ni, ~65 wt% on silica/alumina (Agilent)) was equipped for the detection of CO and CO_2_. To avoid the oxidation of the methanizer, a valve was connected and adjusted by a program for the ventilation of evolved oxygen. For the isotope study, the gas samples were analyzed by GC–MS (Agilent 7890 A/5977B) equipped with a HP-5MS (Agilent) capillary column.

### Data availability

The data that support the findings of this study are available from K.M.N. (email: namkimin.chem@gmail.com) or H.S. (email: hsong@kaist.ac.kr) upon reasonable request.

## Electronic supplementary material

Supplementary Information

Peer Review File

## References

[1] Kondratenko EV, Mul G, Baltrusaitis J, Larrazábal GO, Pérez-Ramírez J (2013). Status and perspectives of CO_2_ conversion into fuels and chemicals by catalytic, photocatalytic and electrocatalytic processes. Energy Environ. Sci..

[2] Song C (2006). Global challenges and strategies for control, conversion and utilization of CO_2_ for sustainable development involving energy, catalysis, adsorption and chemical processing. Catal. Today.

[3] Centi G, Perathoner S (2009). Opportunities and prospects in the chemical recycling of carbon dioxide to fuels. Catal. Today.

[4] Roy SC, Varghese OK, Paulose M, Grimes CA (2010). Toward solar fuels: Photocatalytic conversion of carbon dioxide to hydrocarbons. ACS Nano.

[5] Chang X, Wang T, Gong J (2016). CO_2_ photo-reduction: insights into CO_2_ activation and reaction on surfaces of photocatalysts. Energy Environ. Sci..

[6] Hoffmann MR, Martin ST, Choi W, Bahnemann DW (1995). Environmental applications of semiconductor photocatalysis. Chem. Rev..

[7] Ma Y (2014). Titanium dioxide-based nanomaterials for photocatalytic fuel generations. Chem. Rev..

[8] Habisreutinger SN, Schmidt-Mende L, Stolarczyk JK (2013). Photocatalytic reduction of CO_2_ on TiO_2_ and other semiconductors. Angew. Chem. Int. Ed..

[9] Indrakanti VP, Kubicki JD, Schobert HH (2009). Photoinduced activation of CO_2_ on Ti-based heterogeneous catalysts: Current state, chemical physics-based insights and outlook. Energy Environ. Sci..

[10] Ge M (2016). A review of one-dimensional TiO_2_ nanostructured materials for environmental and energy applications. J. Mater. Chem. A.

[11] Wang W-N (2012). Size and structure matter: Enhanced CO_2_ photoreduction efficiency by size-resolved ultrafine Pt nanoparticles on TiO_2_ single crystals. J. Am. Chem. Soc..

[12] Matsuoka M (2007). Photocatalysis for new energy production: Recent advances in photocatalytic water splitting reactions for hydrogen production. Catal. Today.

[13] Sun S (2015). Recent advances in hybrid Cu_2_O-based heterogeneous nanostructures. Nanoscale.

[14] Park JC, Kim J, Kwon H, Song H (2009). Gram-scale synthesis of Cu_2_O nanocubes and subsequent oxidation to CuO hollow nanostructures for lithium-ion battery anode materials. Adv. Mater..

[15] In S-I, Vaughn DD, Schaak RE (2012). Hybrid CuO-TiO_2-x_N_x_ hollow nanocubes for photocatalytic conversion of CO_2_ into methane under solar irradiation. Angew. Chem. Int. Ed..

[16] Xu H (2014). Porous-structured Cu_2_O/TiO_2_ nanojunction material toward efficient CO_2_ photoreduction. Nanotechnology.

[17] Zaera F (2013). Nanostructured materials for applications in heterogeneous catalysis. Chem. Soc. Rev..

[18] Deo M (2012). Cu_2_O/ZnO hetero-nanobrush: hierarchical assembly, field emission and photocatalytic properties. J. Mater. Chem..

[19] Ren ST, Fan GH, Liang ML, Wang Q, Zhao GL (2014). Electrodeposition of hierarchical ZnO/Cu_2_O nanorod films for highly efficient visible-light-driven photocatalytic applications. J. Appl. Phys..

[20] Wu H, Zhang N, Cao Z, Wang H, Hong S (2012). The adsorption of CO_2_, H_2_CO_3_, HCO_3_^−^ and CO_3_^2−^ on Cu_2_O (111) surface: First-principles study. Int. J. Quantum Chem..

[21] Liu L, Zhao C, Li Y (2012). Spontaneous dissociation of CO_2_ to CO on defective surface of Cu(I)/TiO_2-*x*_ nanoparticles at room temperature. J. Phys. Chem. C.

[22] Park JC (2012). ZnO-CuO core-branch nanocatalysts for ultrasound-assisted azide-alkyne cycloaddition reactions. Chem. Commun..

[23] Game O (2012). Concurrent synthetic control of dopant (nitrogen) and defect complexes to realize broadband (UV-650 nm) absorption in ZnO nanorods for superior photo-electrochemical performance. J. Mater. Chem..

[24] Jézéquel D, Guenot J, Jouini N, Fiévet F (1995). Submicrometer zinc oxide particles: Elaboration in polyol medium and morphological characteristics. J. Mater. Res..

[25] Liu L, Li Y (2014). Understanding the reaction mechanism of photocatalytic reduction of CO_2_ with H_2_O on TiO_2_-based photocatalysts: A review. Aerosol Air Qual. Res.

[26] Zhang Q, Dandeneau CS, Zhou X, Cao G (2009). ZnO nanostructures for dye-sensitized solar cells. Adv. Mater..

[27] Li Y (2009). Comparison of dye photodegradation and its coupling with light-to-electricity conversion over TiO_2_ and ZnO. Langmuir.

[28] Jiang T, Xie T, Chen L, Fu Z, Wang D (2013). Carrier concentration-dependent electron transfer in Cu_2_O/ZnO nanorod arrays and their photocatalytic performance. Nanoscale.

[29] Kim W, Seok T, Choi W (2012). Nafion layer-enhanced photosynthetic conversion of CO_2_ into hydrocarbons on TiO_2_ nanoparticles. Energy Environ. Sci..

[30] Sasikala R (2008). Enhanced photocatalytic hydrogen evolution over nanometer sized Sn and Eu doped titanium oxide. Int. J. Hydrog. Energy.

[31] Wang Y, Li S, Shi H, Yu K (2012). Facile synthesis of p-type Cu_2_O/n-type ZnO nano-heterojunctions with novel photoluminescence properties, enhanced field emission and photocatalytic activities. Nanoscale.

[32] Musselman KP (2011). A novel buffering technique for aqueous processing of zinc oxide nanostructures and interfaces, and corresponding improvement of electrodeposited ZnO-Cu_2_O photovoltaics. Adv. Funct. Mater..

[33] Zhou P, Yu J, Jaroniec M (2014). All-solid-state Z-scheme photocatalytic systems. Adv. Mater..

[34] Wang J-C (2015). Enhanced photoreduction CO_2_ activity over direct Z-scheme α-Fe_2_O_3_/Cu_2_O heterostructures under visible light irradiation. ACS Appl. Mater. Interfaces.

[35] Wang Q (2016). Scalable water splitting on particulate photocatalyst sheets with a solar-to-hydrogen energy conversion efficiency exceeding 1%. Nat. Mater.

[36] Zhang Y-G, Ma L-L, Li J-L, Yu Y (2007). In situ fenton reagent generated from TiO_2_/Cu_2_O composite film: a new way to utilize TiO_2_ under visible light irradiation. Environ. Sci. Technol..

[37] Nozik AJ (1977). Photochemical diodes. Appl. Phys. Lett..

[38] Walter MG (2010). Solar water splitting cells. Chem. Rev..

[39] Wang W-N (2015). Surface engineered CuO nanowires with ZnO islands for CO_2_ photoreduction. ACS Appl. Mater. Interfaces.

[40] Kim D, Resasco J, Yu Y, Asiri AM, Yang P (2014). Synergistic geometric and electronic effects for electrochemical reduction of carbon dioxide using gold-copper bimetallic nanoparticles. Nat. Comm.

[41] Gattrell M, Gupta N, Co A (2006). A review of the aqueous electrochemical reduction of CO_2_ to hydrocarbons at copper. J. Electronal. Chem.

[42] Peterson AA, Abild-Pedersen F, Studt F, Rossmeisl J, Nørskov JK (2010). How copper catalyzes the electroreduction of carbon dioxide into hydrocarbon fuels. Energy Environ. Sci..

[43] Cox DF, Schulz KH (1991). Interaction of CO with Cu^+^ cations: CO adsorption on Cu_2_O(100). Surf. Sci.

[44] Eggins, B. R., Irvine, J. T. S., Murphy, E. P. & Grimshaw, J. Formation of two-carbon acids from carbon dioxide by photoreduction on cadmium sulphide. *J. Chem. Soc. Chem. Commun*. **0**, 1123–1124 (1988).

[45] Wang Z-Y, Chou H-C, Wu JCS, Tsai DP, Mul G (2010). CO_2_ photoreduction using NiO/InTaO_4_ in optical-fiber reactor for renewable energy. Appl. Catal. A-Gen..

[46] Tseng I-H, Chang W-C, Wu JCS (2002). Photoreduction of CO_2_ using sol-gel derived titania and titania-supported copper catalysts. Appl. Catal. B-Environ..

[47] Maidan R, Willner I (1986). Photoreduction of CO_2_ to CH_4_ in aqueous solutions using visible light. J. Am. Chem. Soc..

[48] Neaţu Ş, Maciá-Agulló JA, Concepción P, Garcia H (2014). Gold-copper nanoalloys supported on TiO_2_ as photocatalysts for CO_2_ reduction by water. J. Am. Chem. Soc..

