# Supplementary material for: Colloidal zinc oxide-copper(I) oxide nanocatalysts for selective aqueous photocatalytic carbon dioxide conversion into methane
Source: Nat Commun. 2017 Nov 7;8:1156. doi: 10.1038/s41467-017-01165-4 (PMC5673890; doi:10.1038/s41467-017-01165-4)
Supplement: Supplementary file 1 — Supplementary Information [file 41467_2017_1165_MOESM1_ESM.pdf]

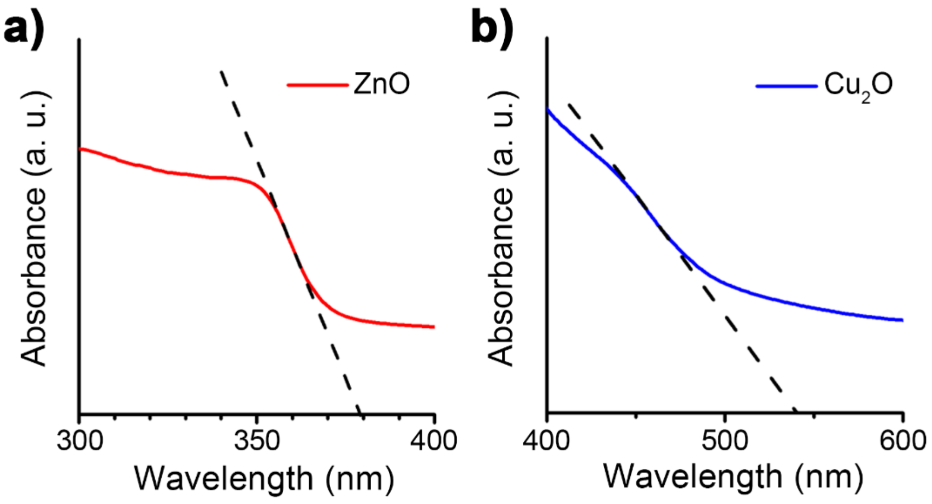

Supplementary Figure 1 | UV-Vis spectra and Tauc plots of (a) ZnO and (b) Cu_2O nanoparticles.

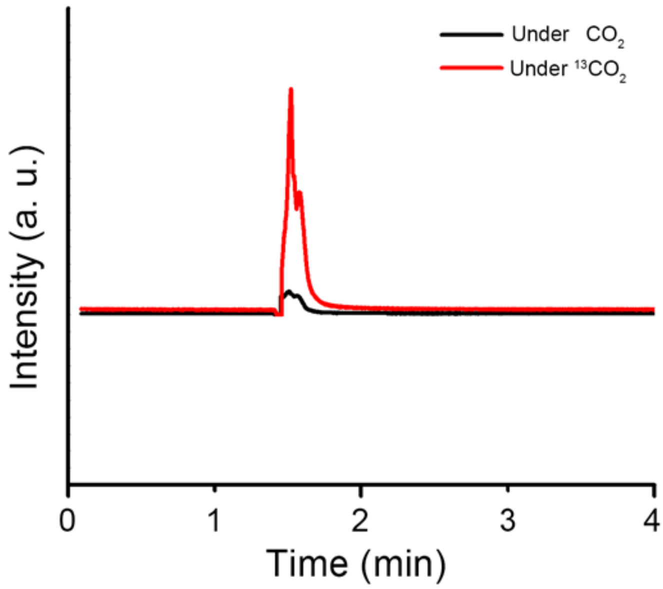

Supplementary Figure 2 | GC-MS chromatogram at m/e = 17 using $^{13}CO_2$ and $Na_2{}^{13}CO_3$ as carbon sources for CO_2 reduction.

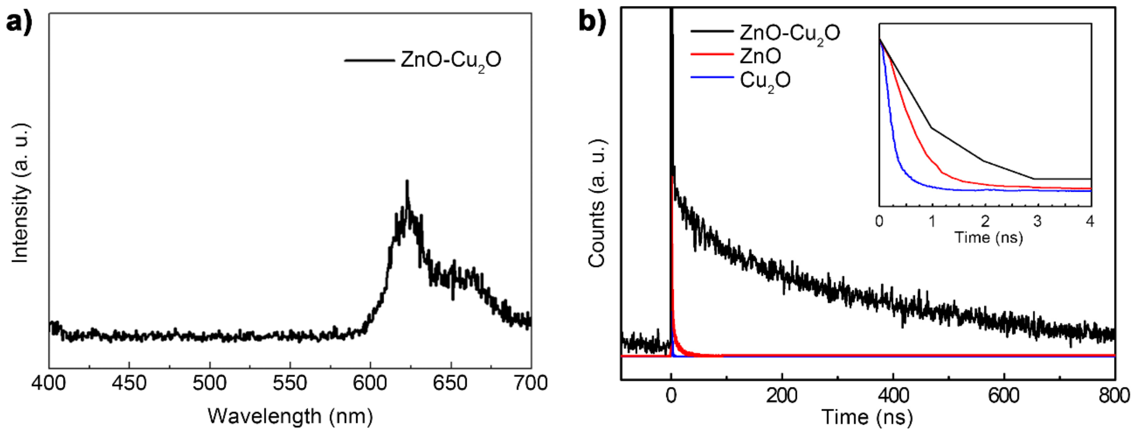

Supplementary Figure 3 | (a) Emission profile of the ZnO-Cu_2O hybrid nanoparticles irradiated by the laser light at the wavelength of 375 nm. (b) Transient absorption spectra at 621 nm on ZnO-Cu_2O catalysts (black), and ZnO (red) and Cu_2O (blue) nanoparticles by the irradiation of laser light (λ = 375 nm).

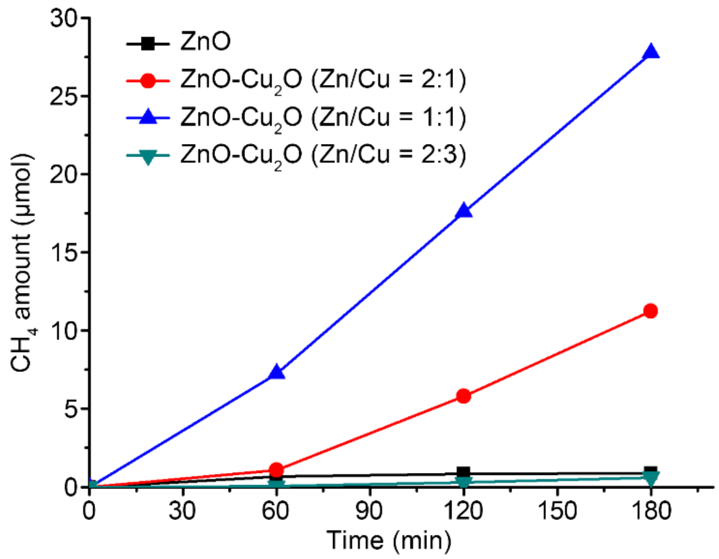

Supplementary Figure 4 | CH_4 production using the catalysts with different Zn/Cu ratios. Amount of CH_4 production using the ZnO-Cu_2O catalysts synthesized from the Zn and Cu precursor ratios of 2:1 (red), 1:1 (blue, optimized structure), and 2:3 (green), and using pure ZnO aggregates (black).

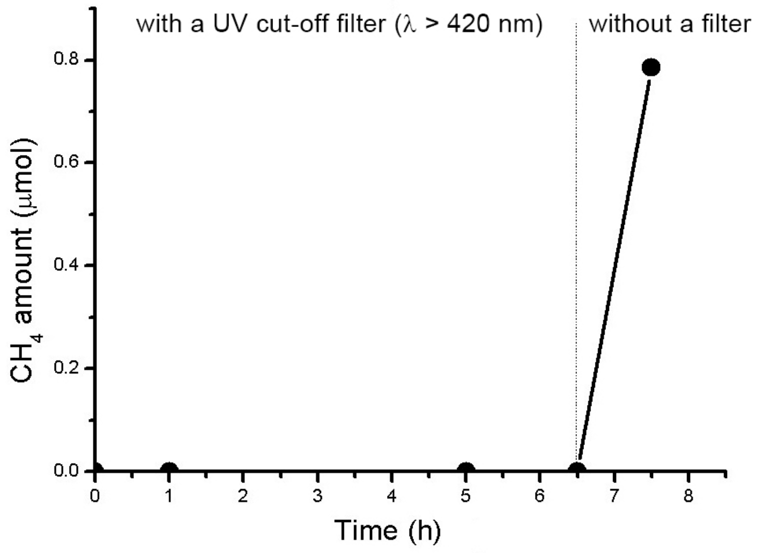

Supplementary Figure 5 | Photocatalytic reactions by the irradiation of visible light. Amount of CH_4 production under the irradiation of visible light with a UV cut-off filter (λ > 420 nm). After 6.5 h irradiation, the filter was removed. The light intensity before and after the removal of the filter was fixed at 0.59 Wcm^{-2} by adjusting the distance between the light source and the reactor.

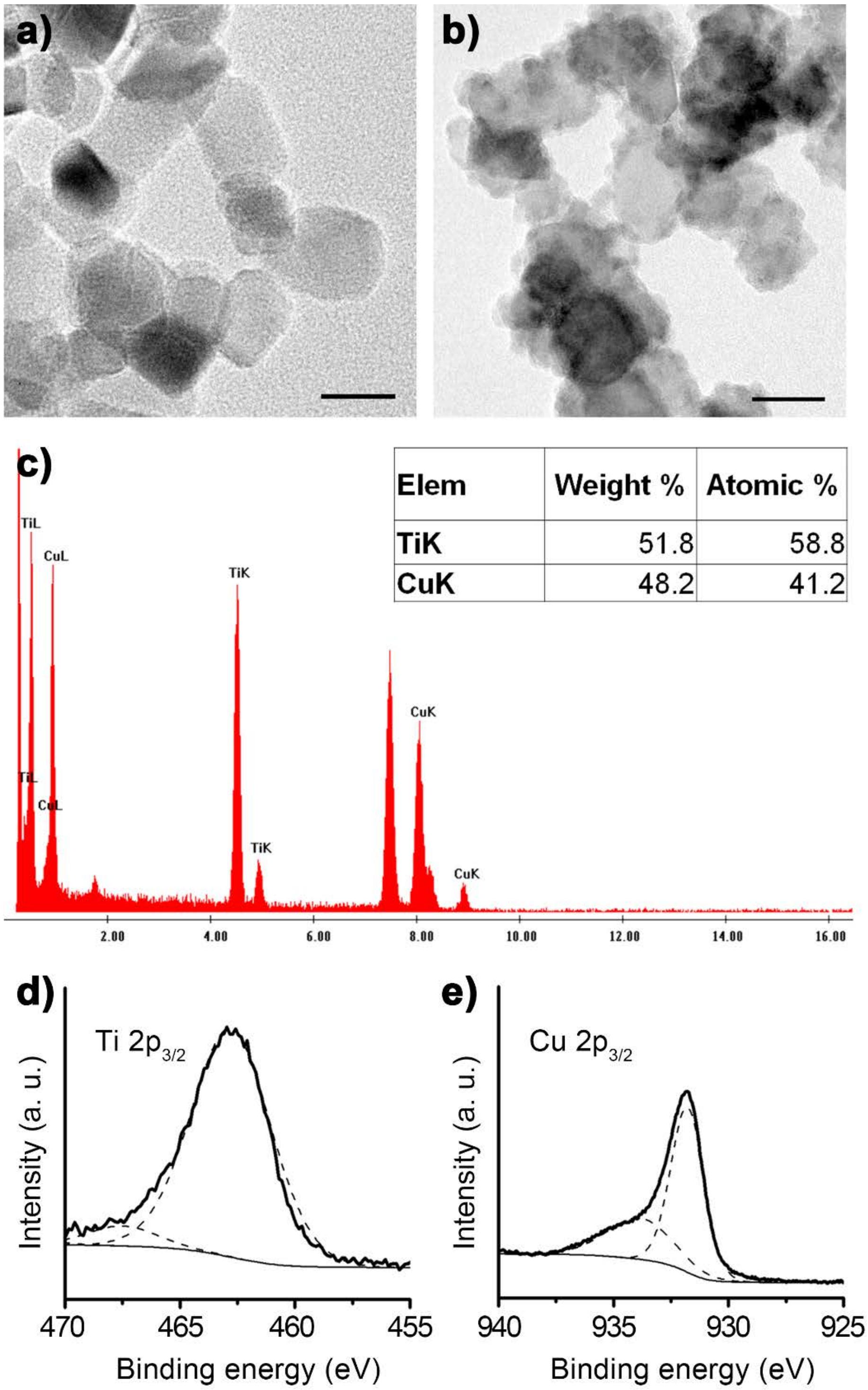

Elem	Weight %	Atomic %
TiK	51.8	58.8
CuK	48.2	41.2

Supplementary Figure 6 | TEM images of (a) commercial P25 and (b) a TiO_2-Cu_2O hybrid structure. The bars represent 20 nm. (c) EDX spectrum the TiO_2-Cu_2O hybrid structure. XPS spectra of the TiO_2-Cu_2O hybrid structure in the regions of (d) Ti $2p_{3/2}$ and (e) Cu $2p_{3/2}$.

Supplementary Notes

Supplementary Note 1: Calculation of Quantum Efficiency

The quantum efficiency was calculated using a method reported by Bharadwaj et al.[1] To measure the incident light power, the power meter (Newport 848-R) was located at the center where the reactor was placed during the reaction. The light power was corrected in consideration of the difference between the detector area of the power meter and the irradiation area of the reactor with the assumption of uniform distribution of the light intensity. The effective absorption for the photocatalytic reaction was in the range of 200-540 nm, based on the UV-Vis absorption of the ZnO-Cu_2O catalysts. To calculate the fraction of the light power at each wavelength, the emission profile of the Xe lamp was measured using a fiber-coupled spectrometer (Ocean Optics USB4000) in the range of 200-700 nm. The light power in the range of 200-540 nm (E_m) was calculated by multiplying the total light power measured in the range of 200-700 nm with the fraction of the area (200-540 nm) to the total area (200-700 nm) in the emission spectrum. The energy of a single photon was estimated to be the weighted average energy of all photons in the wavelengths of 200-700 nm.

The weighted average energy (E_a) of a single photon is calculated by the following equations:

$$\boldsymbol{E_a = \sum_{\lambda_i=200}^{520} E_i(\lambda_i)}$$

$$\boldsymbol{E_i(\lambda_i) = \frac{I_{\lambda_i}}{I_{total}} \times \frac{hc}{\lambda_i}}$$

where λ_i, $I_{\lambda i}$ and I_{total} are the wavelength at i (i varying from 200 nm to 540 nm), the intensity at the wavelength λ_i and the total intensity of the emission spectrum of the Xe lamp in the region of 200-540 nm, respectively.

The number of incident photons in the range of 200-540 nm is calculated by the following equation:

$$\boldsymbol{N = \frac{E_m}{E_a}}$$

Supplementary Note 2: Calculation of CO_2 Conversion

Total CO_2 conversion was estimated based on the amount of dissolved CO_2 in water using the following equations:[2,3]

$$\text{Conversion (\%)} = \frac{The\ amount\ of\ generated\ CH_4\ molecules\ (mol)}{The\ amount\ of\ CO_2\ molecules\ in\ water\ (mol)} \times 100$$

$$CO_2 + H_2O \rightleftharpoons H^+ + HCO_3^- \quad (1)$$

$$HCO_3^- \rightleftharpoons H^+ + CO_3^{2-} \quad (2)$$

$$[CO_2]_{eq} = \frac{[H^+]_{eq}^2}{[H^+]_{eq}^2 + K_1[H^+]_{eq} + K_1K_2} \times DIC$$

$$[HCO_3^-]_{eq} = \frac{K_1[H^+]_{eq}}{[H^+]_{eq}^2 + K_1[H^+]_{eq} + K_1K_2} \times DIC$$

$$[CO_3^{2-}]_{eq} = \frac{K_1K_2}{[H^+]_{eq}^2 + K_1[H^+]_{eq} + K_1K_2} \times DIC$$

$$\text{DIC (Total dissolved inorganic carbon)} = \left[CO_{2(aq)}\right] + [HCO_3^-] + [CO_3^{2-}]$$

When the total amount of CO_2 including dissolved and gaseous forms participates in the reaction, the CO_2 conversion is estimated as 41% after the 14 h irradiation of light. In the actual experiment, the CO_2 concentration dissolved in water may further decrease due to the temperature rising by prolonged irradiation. Severe decrease of the reactivity due to the transfer of dissolved CO_2 to the gas phase was also reported in the literature.[4]

Supplementary References

1. Sasikala, R. *et al.* Enhanced photocatalytic hydrogen evolution over nanometer sized Sn and Eu doped titanium oxide. *Int. J. Hydrog. Energy* **33,** 4966-4973 (2008).

2. Barker, S. & Ridgwell, A. Ocean acidification. *Nature Education Knowledge* 3, 21 (2012).

3. Wolf-Gladrow, D. A., Zeebe, R. E., Klaas, C., Kortzinger, A. & Dickson, A. G. Total alkalinity: The explicit conservative expression and its application to biogeochemical processes. *Marine Chem.* 106, 287-300 (2007).

4. Kim, W., Seok, T. & Choi, W. Nafion layer-enhanced photosynthetic conversion of CO_2 into hydrocarbons on TiO_2 nanoparticles. *Energy Environ. Sci.* **5**, 6066-6070 (2012).
